# Sample Buffer Containing Guanidine-Hydrochloride Combines Biological Safety and RNA Preservation for SARS-CoV-2 Molecular Diagnostics

**DOI:** 10.3390/diagnostics12051186

**Published:** 2022-05-10

**Authors:** Lisa Weidner, Sandra Laner-Plamberger, David Horner, Charlotte Pistorius, Jennifer Jurkin, Michael Karbiener, Elisabeth Schistal, Thomas R. Kreil, Christof Jungbauer

**Affiliations:** 1 Austrian Red Cross, Blood Service for Vienna, Lower Austria and Burgenland, Wiedner Hauptstraße 32, 1040 Vienna, Austria; lisa.weidner@roteskreuz.at (L.W.); david.horner@roteskreuz.at (D.H.); charlotte.pistorius@roteskreuz.at (C.P.); jennifer.jurkin@roteskreuz.at (J.J.); elisabeth.schistal@roteskreuz.at (E.S.); 2 Department for Transfusion Medicine, University Hospital of Salzburg (SALK), Paracelsus Medical University (PMU), Müllner-Hauptstraße 48, 5020 Salzburg, Austria; s.laner-plamberger@salk.at; 3 Spinal Cord Injury and Tissue Regeneration Centre Salzburg, PMU Salzburg, Strubergasse 21, 5020 Salzburg, Austria; 4 Global Pathogen Safety, Takeda Manufacturing Austria AG, Benatzkygasse 2-6, 1221 Vienna, Austria; michael.karbiener@takeda.com (M.K.); thomas.kreil@takeda.com (T.R.K.)

**Keywords:** virus inactivation, sample storage buffer, RNAse activity, SARS-CoV-2

## Abstract

The COVID-19 pandemic has elicited the need to analyse and store large amounts of infectious samples for laboratory diagnostics. Therefore, there has been a demand for sample storage buffers that effectively inactivate infectious viral particles while simultaneously preserving the viral RNA. Here, we present a storage buffer containing guanidine-hydrochloride that fulfils both requirements. Its ability to preserve RNA stability was confirmed by RT-qPCR, and virus-inactivating properties were tested by tissue culture infectious dose assay. Our data revealed that RNA from samples diluted in this storage buffer was efficiently preserved. Spiking samples with RNase A resulted in RNAse concentrations up to 100 ng/mL being efficiently inhibited, whereas spiking samples with infectious SARS-CoV-2 particles demonstrated rapid virus inactivation. In addition, our buffer demonstrated good compatibility with several commercially available RNA extraction platforms. The presented guanidine-hydrochloride-based storage buffer efficiently inactivates infectious SARS-CoV-2 particles and supports viral RNA stability, leading to a reduced infection risk during sample analysis and an increased period for follow-up analysis, such as sequencing for virus variants. Because the presented buffer is uncomplicated to manufacture and compatible with a variety of commercially available test systems, its application can support and improve SARS-CoV-2 laboratory diagnostics worldwide.

## 1. Introduction

In December 2019, a respiratory illness emerged in China, which quickly spread globally and was declared a pandemic in March 2020 by the World Health Organization (WHO) [1]. Severe acute respiratory syndrome coronavirus 2 (SARS-CoV-2) was identified as the causative agent of this illness, which was termed coronavirus disease 2019 (COVID-19) [2]. Although vaccination has been available since the beginning of 2021, SARS-CoV-2 virus variants are fast-spreading around the globe, causing new infections and reinfections every day [3].

To date, more than 500 million infections have been documented (https://coronavirus.jhu.edu/map.html, 13 April 2022), challenging already seriously affected health care systems not only due to the immense level of care necessary for many patients but also the diagnostic work of laboratories, as large numbers of samples need to be processed in a short period of time. There are two major issues as far as laboratory diagnostics are concerned. First, the conservation of genetic material from patient samples is a key step for reliable and consistent molecular diagnostics [4,5]. Degradation or fragmentation of genetic material often occurs due to specific enzymes (DNases/RNases), which may also be introduced exogenously [6]. RNA stability is a major concern for SARS-CoV-2 diagnostics, as RNA degradation or fragmentation may increase the rate of false-negative results, making it difficult to fight virus dissemination. Thus, it is important to take suitable precautions to inhibit and avoid nucleic acid degradation that do not influence the performance of the diagnostic methodology used.

The second major issue in relation to laboratory diagnostics is virus inactivation [7,8]. Efficient viral inactivation, which is important in the transportation and analysis of contaminated samples, depends on several factors, such as the type of pathogen, its concentration, the sample matrix, the type and concentration of the inactivation agent applied and the contact time [8]. To date, data regarding inactivation of viral infectivity and RNA stability of SARS-CoV-2 samples are rare [9,10]. Welch et al. evaluated 23 different commercially available sampling solutions, demonstrating that the majority of reagents lead to a reduction in the viral load. Nevertheless, some of the most commonly used solutions showed remaining infectivity [8]. As reviewed by Alturki et al., the main routes of SARS-CoV-2 transmission are person-to-person contact, aerosols, and contaminated hands or surfaces [11]. Staff of diagnostic laboratories performing SARS-CoV-2 diagnostics are at risk when working with infectious human material. Therefore, efficient viral inactivation while also ensuring RNA stability is urgently required.

The aim of this study was to develop a sample storage buffer (SSB) that is uncomplicated to manufacture, compatible with commercially available RT-qPCR test systems, allows for safe long-term sample storage preserving RNA stability and simultaneously and efficiently reduces the infectivity of samples.

## 2. Materials and Methods

### 2.1. Sample Storage Buffers

For this study, two in-house manufactured, guanidine-hydrochloride-containing storage sample buffers, SSB-4M and SSB-6M, were compared to 0.9% NaCl (control) and two commercially available sample buffers: DNA/RNA Shield from Zymo (Zymo, Irvine, CA, USA) and Cobas PCR media from Roche (Roche, Diagnostics, Rotkreuz, Switzerland). Table 1 shows the chemical composition of the in-house buffers, SSB-4M and SSB-6M.

### 2.2. Sample Collection

All participants signed an informed consent, and samples were processed anonymously to protect participants’ privacy. Samples were collected from 18 individuals who had previously tested SARS-CoV-2-positive by RT-qPCR 5–7 days after symptom onset. Oro- and nasopharyngeal swabs were taken and washed in SSB-4M sample buffer. For pharyngeal lavage samples, participants were asked to gargle with 5 mL of 0.9% NaCl for 60 s. The final lavage sample was diluted 1:1 in SSB-4M. RNA was extracted and subjected to RT-qPCR immediately using a Roche Cobas 8800 test system and a Promega LEV Buccal Swab Maxwell kit. To assess ribonuclease activity, pharyngeal lavage samples from SARS-CoV-2-negative individuals were collected and pooled to avoid donor-specific effects.

### 2.3. Sample Spiking

For spiking with intact virus particles, pharyngeal lavage from one individual with a high viral load (Ct value: 18) was used to spike samples at dilutions of 1:50 (=mock swab samples). Additionally, SARS-CoV-2 RNA was isolated from a sample of the same individual, applying the Zymo Viral DNA/RNA MagBead kit, and used for RNA spiking experiments. RNase A (Omega Bio-Tek, 100 mg/mL solution) was used to spike different sampling solutions. A dilution series of RNase A (1, 10 and 100 ng/mL; 1 and 10 µg/mL) in SSB-4M (undiluted and using a 1:1 dilution with 0.9% NaCl) was performed to determine the concentration-dependent inactivation of RNase A. All samples used were spiked with infectious SARS-CoV-2 particles at a dilution of 1:50 and analysed for up to 14 days. Additionally, TBS buffer (0.9% NaCl, 50 mM Tris pH 8.5) without any detergents, with 0.1% Tween or 0.5% Triton X was spiked with RNAse A at different concentrations (0.001, 0.1 and 1 pg/mL) and SARS-CoV-2 RNA. Spiked samples were subjected to RT-qPCR immediately, as well as 10, 20 and 30 min post-spiking.

### 2.4. RNA Extraction

Unless otherwise stated, RNA was extracted in a 96-deepwell plate (Roche, Diagnostics, Rotkreuz, Switzerland) using a Zymo Viral DNA/RNA MagBead kit with a modification: 10 mM DDT was added to the viral DNA/RNA buffer. Other commercially available RNA extraction kits used in this study were the Qiagen Viral RNA mini kit (Qiagen, Hilden, Germany), the Promega LEV buccal swab kit (Promega Corp, Madison, Wisconsin) and the Roche Cobas 8800 SARS-CoV-2 kit (Roche, Diagnostics, Rotkreuz, Switzerland). All kits were used according to manufacturer’s instructions. In addition, an in-house RNA extraction method was performed as follows: 200 µL sample was incubated with 0.25 mg/mL proteinase K (Zymo Research, Irvine, CA, USA) for 20 min at 50 °C. Subsequently, RNA was captured in 10 µL of a silica magnetic bead solution (100 mg/mL buffered in 4 M GuHCl and 50 mM Tris; pH: 6.5) and 400 µL binding buffer (5 M GuHCl, 120 mM sodium acetate (pH 5.2), 0.1% (*v*/*v*) Tween-20, 40% 2-Propanol). Samples were pipetted into a 96-well plate, placed on a magnetic holder (PerkinElmer chemagen Technologie GmbH, 52499 Baesweiler, Germany) and washed twice with Wash Buffer 1 (5 M GuHCl, 20 mM Tris-HCl (pH 6.5), 40% 2-Propanol), once with Wash Buffer 2 (20 mM Tris-HCl (pH 6.5), 60% 2-Propanol) and twice with absolute ethanol. Silica magnetic beads were air-dried, and finally, bound RNA was eluted in 20 µL of DNase/RNase-free water. The eluate was directly used for further analysis.

### 2.5. Real-Time RT-PCR

Unless otherwise stated, RT-qPCR was conducted by applying a CFX96 Touch real-time PCR detection system (Biorad, Hercules, CA, USA) using 2.5 µL of the eluate, 2.5 µL Thermofisher TaqPath™ 1-Step RT-qPCR master mix (Thermo Fisher Scientific, Rochester, NY, USA) and 0.25 µL LightMix Modular Wuhan CoV RdRP-gene primer mix (cat-No. 53-0777-96, Tib Molbiol, Berlin, Germany) in a final reaction volume of 10 µL. An initial step of reverse transcription at 50 °C for 10 min followed by Taq activation at 95 °C for 5 min and 45 cycles of 95 °C 5 s, 60 °C 15 s and 72°C 15 s was performed.

### 2.6. Virus Propagation and Titration

SARS-CoV-2 and Vero cells were used as previously described [12]. For titration of virus infectivity, median tissue culture infectious dose (TCID50) assays were performed in eightfold technical replicates of serial half log10 sample dilutions. Virus-containing samples were incubated at 36 °C under 5% CO_2_ on Vero cells seeded in 96-well plates. After 5–7 days of incubation, cytopathic effects were evaluated by microscopic visual inspection. TCID50 titres were calculated according to the Poisson distribution and expressed as log10 (TCID50/mL). Virus reduction factors were calculated in accordance with the EU Committee for Proprietary Medicinal Products guidance [13].

### 2.7. Size-Exclusion Chromatography

To separate potential cytotoxic substances, such as Triton X-100 and GuHCl, samples were subjected to size-exclusion chromatography (SEC) using a PD-10 protein desalting column (GE Healthcare, Chicago, IL, USA) containing 8.3 mL of SephadexTM G-25 medium. Prior to use, column equilibration was performed using equilibration buffer (0.1% human serum albumin in 1xPBS) according to manufacturer’s instructions. After equilibration, 2.5 mL samples were subjected to the PD-10 desalting column. The virus-containing fraction was eluted by centrifugation (1000× *g*, 2 min, 17 ± 1 °C).

### 2.8. Viral Inactivation Assay

SSB was diluted to 1:10 with either 0.9% NaCl or EDTA/TRIS buffer (0.74% EDTA and 0.6% TRIS). A separate vessel containing 0.9% NaCl or only EDTA/TRIS buffer was used as a control representing a matrix without virus-inactivating compounds. During experimental runs, the material was kept at 17 ± 1 °C and continuously mixed by a magnetic stirrer. Spiking with virus stock solution was performed at a ratio of 1:11. Spike control (SC) and hold control (HC) were drawn from the control vessel and divided into two parts. The first part was titrated immediately, whereas the second part was subjected to SEC prior to titration. SC was drawn 2 min after virus spike, whereas HC was drawn 60 ± 1 min after virus spike. Samples for TCID50 virus titration were drawn from the vessel containing 10% stabilizing solution at 2 min, 5 ± 1 min, 10 ± 1 min, 30 ± 1 min and 60± 1 min after virus spike. To minimize further inactivation of the virus, the samples were immediately subjected to SEC prior to titration. In order to lower the limit of detection for samples from the final fractions, larger volumes were titrated in addition to the regular TCID50 assay by applying selected sample dilutions (i.e., undiluted, 0.5 log10-diluted, 1 log10-diluted and 1.5 log10-diluted) not to a single column (i.e., 8 wells) but to all 96 wells of a microtiter plate (‘bulk titration’). The cytotoxicity of the stabilizing solution, as well as any possible matrix effects on cell lines used for virus detection, were tested and taken into account for the calculation of reduction factors.

### 2.9. Statistics

Results were statistically analysed using unpaired Student’s *t*-test. A *p*-value < 0.05 was considered statistically significant.

## 3. Results

### 3.1. Sample Storage Buffer SSB-4M Is Compatible with Commercially Available and In-House RNA Extraction Methods

First, we tested the compatibility of the storage sample buffer containing 4M GuHCl (SSB-4M) with three different commercially available extraction methods (Qiagen Viral RNA mini kit, Promega LEV buccal swab kit and Zymo Viral DNA/RNA MagBead kit) and our in-house RNA extraction method. In order to do so, we extracted viral RNA from SARS-CoV-2-spiked SSB-4M buffer. For RNA isolation, we either used lysis buffer for the extraction method diluted with SSB-4M or 0.9% NaCl as control (1:1) or SSB-4M as lysis buffer only. RT-qPCR revealed comparable cycle threshold (Ct) values for all lysis buffers used (Table 2).

We also tested the compatibility of the SSB-4B buffer with the fully automated Cobas 8800 platform from Roche diagnostics, comparing undiluted SSB-4M to the Roche PCR media buffer. Again, the Ct values were similar: 27.47 +/− 0.12 Ct for SARS-CoV-2 target 1 and 27.47 +/− 0.12 Ct for SARS-CoV-2 target 2 using SSB 4M and 27.75 +/− 0.12 Ct (SARS-CoV-2 target 1) and 27.85 +/− 0.22 Ct (SARS-CoV-2 target 2) for the Roche PCR media buffer. Our data demonstrate the compatibility of SSB-4M with commercially available and in-house GuHCl-based RT-qPCR test systems.

### 3.2. SARS-CoV-2 RNA Is Stable in Swab Samples Stored in SSB-4M, Even in the Presence of RNAse A

**Figure 1:**
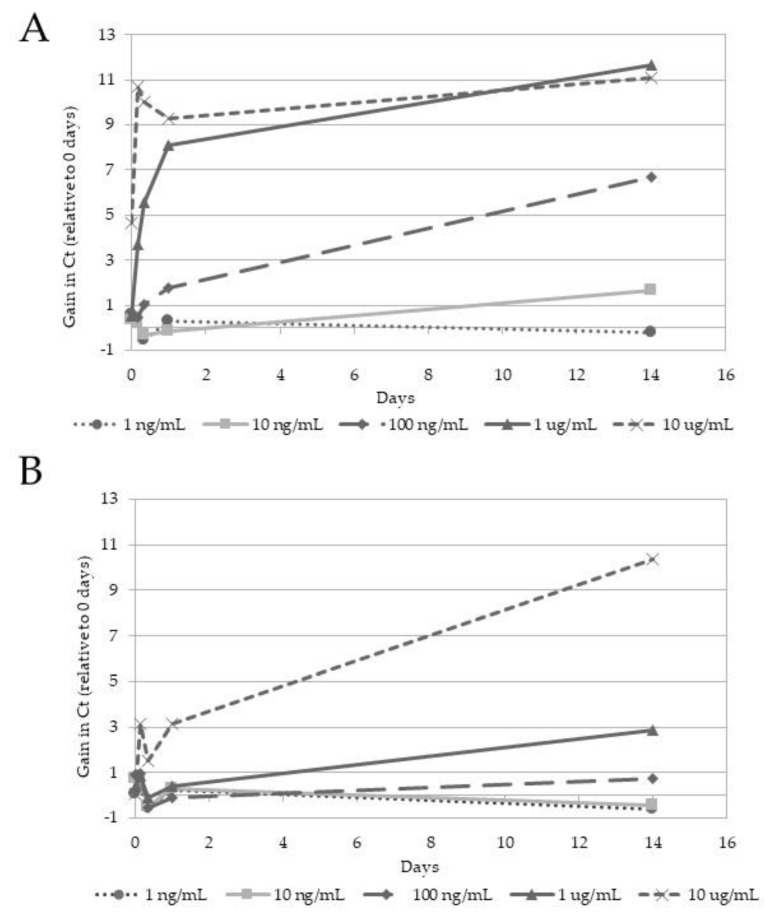
SARS-CoV-2 RNA stability of swab samples in SSB-4M in the presence of RNase A. RNA was isolated from samples in SSB-4M diluted to 1:1 with 0.9% NaCl (**A**) or undiluted SSB-4M (**B**) spiked with SARS-CoV-2 viral particles. Different concentrations of RNase A were added as indicated, and the presence of SARS-CoV-2 RNA was measured at five different points in time (0, 4, 8, 24 h and 14 days post-RNAse A spiking). The relative gain of Ct is shown over time.

As a next step, we spiked SARS-CoV-2-containing mock swab samples stored in SSB-4M (1:1 diluted with 0.9% NaCl) with increasing concentrations of RNAse A to investigate the protective effect against RNase degradation at room temperature. As shown in Figure 1A, when spiked with 1 ng/mL RNAse A, the RNA sample was stable for more than two weeks, whereas in the presence of 10 ng/mL RNAse A, the Ct values slightly increased. Concentrations of 100 ng/mL led to an increase of more than 6 Ct. When spiked with 1 µg/mL and 10 µg/mL RNase A, an immediate gain of 8–10 Ct was observed, followed by a continuous increase over time (11 Ct). To sum up, RNase concentrations up to 10 ng/mL were efficiently inhibited by diluted SSB-4M. However, higher concentrations of RNase A (100 ng^−1^ µg/mL) led to a rapid gain in Ct values over time. We also investigated the protective effect of undiluted SSB-4M and found that concentrations of up to 100 ng/mL RNAse A were efficiently inhibited, as no increase in Ct values was observed (Figure 1B).

### 3.3. Different Samples Require Different Storage Buffer Conditions to Efficiently Preserve SARS-CoV-2 RNA

**Figure 2:**
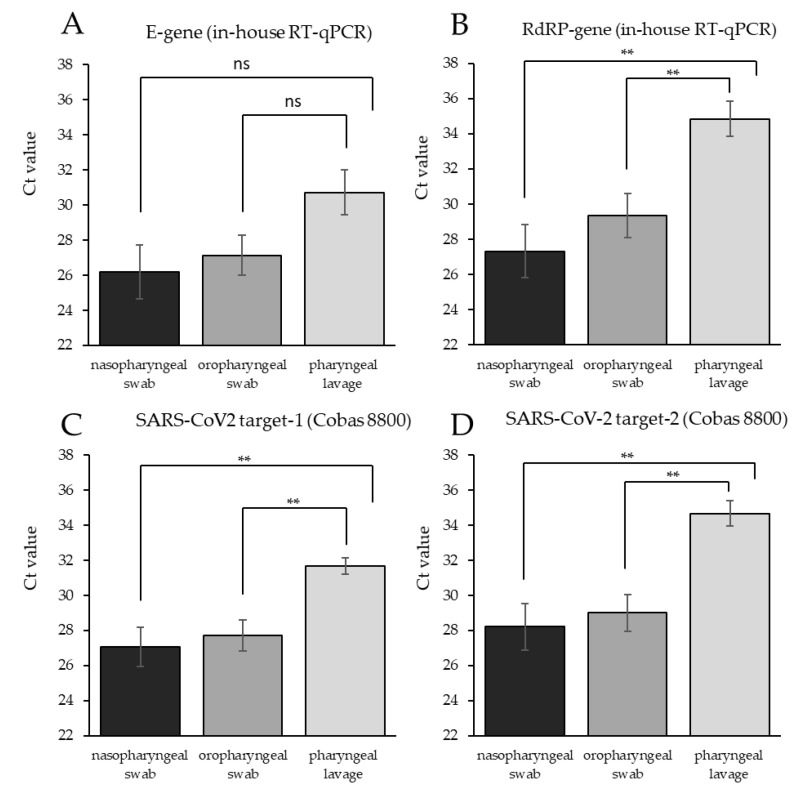
SSB-4M efficiently preserves RNA in naso- and oropharyngeal swab samples, but not in pharyngeal lavage. RNA was isolated from different sample types (naso- or oropharyngeal swabs or pharyngeal lavage). Each sample type was collected from all 16 SARS-CoV-2-positive individuals. In-house RT-qPCR was applied to screen for SARS-CoV-2 E gene (**A**), and the SARS-CoV-2 RdRP gene (**B**). In addition, also Roche Cobas 8800 SARS-CoV-2 target screening was performed (**C**,**D**). Data shown are mean Ct values +/− standard deviation of 16 biological replicates for each sample type. ** *p* < 0.01, ns = not significant.

To test whether SSB-4M can be used for the inactivation and storage of SARS-CoV-2 in different sample types, we compared naso- and oropharyngeal swabs with pharyngeal lavage. All samples were collected from 16 individuals who had previously tested positive for SARS-CoV-2. Swabs were directly stored in SSB-4M, and pharyngeal lavage was diluted with SSB-4M at the point of care. RT-qPCR targeting the SARS-CoV-2 E gene revealed substantially, although not significantly higher, Ct values in pharyngeal lavage samples compared to swab samples (Figure 2A). Furthermore, we found significantly increased Ct values of the SARS-CoV-2 RdRP gene in lavage samples when applying in-house RNA isolation and RT-qPCR (Figure 2B). The same effect was observed when using the Roche Cobas 8800 RNA extraction and RT-qPCR system (Figure 2C,D). No significant difference was observed between naso- and oropharyngeal swab samples, independent of the RNA extraction method and RT-qPCR system applied (Figure 2A,D). It is important to note that pharyngeal lavage showed a higher number of false-negative results compared to naso- and oropharyngeal swabs (Table 3). The Cobas 8800 SARS-CoV-2 RT-qPCR system correctly detected SARS-CoV-2 positivity in 11 out of 16 pharyngeal lavage samples, whereas our in-house screening identified 14 out of 16 positive samples correctly. However, the in-house screening for SARS-CoV-2 E-Gen identified only 6 of 16 pharyngeal lavage samples, indicating that this screening approach is less reliable. Taken together, our data show that the SSB-4M buffer efficiently protects SARS-CoV-2 RNA stability in naso- and oropharyngeal swab samples but not in pharyngeal lavage.

**Figure 3:**
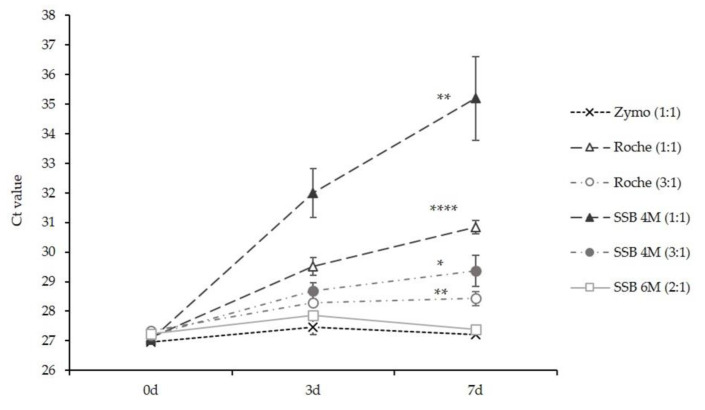
Chaotropic solutions with >4M GuHCl efficiently preserve SARS-CoV-2 RNA stability in pharyngeal lavage. Pharyngeal lavage samples of healthy individuals were diluted in different sample inactivation buffers, as indicated (buffer: pharyngeal lavage). RT-qPCR was performed with RNA isolated at the points in time indicated. Data shown are mean Ct-values +/− standard deviation (* *p* < 0.05, ** *p* < 0.01, **** *p* < 0.0001).

Because SSB-4M does not sufficiently protect RNA stability in pharyngeal lavage samples, we decided to modify SSB-4M and increase the concentration of GuHCl to 6M (=SSB-6M, Table 1). To test RNA stability in SSB-6M, we spiked pharyngeal lavage samples of healthy individuals with intact SARS-CoV-2 viral particles and diluted the samples in SSB-6M (buffer: sample = 2:1), SSB4-M (1:1 and 3:1) and different commercially available storage buffers according to the manufacturer’s instructions. RNA extraction and RT-qPCR were performed immediately after spiking (0 days), as well as 3 and 7 days post-spiking. Comparing Ct values between day 0 and day 7 after spiking, we found sufficient RNA stability for SSB-6M and the commercially available Zymo DNA/RNA Shield buffer (Figure 3). All other storage buffer systems revealed a significant increase in Ct values and thus a significant decrease in SARS-CoV-2 RNA stability.

### 3.4. Tween-20 and Triton X-100 Detergents Decrease the Stability of SARS-CoV-2 RNA

**Figure 4:**
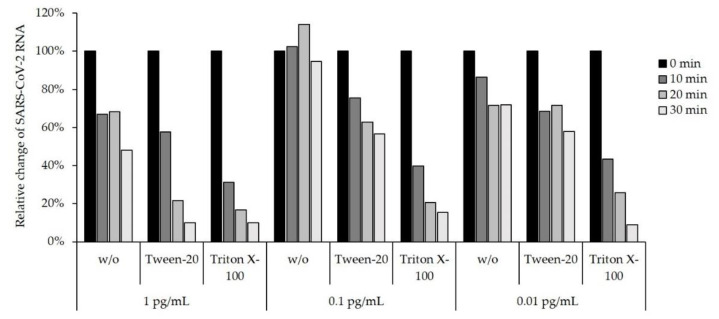
The use of Tween-20 or Triton X-100 detergents decreases the stability of SARS-CoV-2 RNA. TBS buffer was spiked with purified RNA from a SARS-CoV-2-positive individual (control sample, w/o). Furthermore, TBS with 0.1% Tween-20 or TBS with 0.5% Triton X-100 was spiked with purified SARS-CoV-2 RNA. RNase A was added to the spiked buffers at different concentrations as indicated. RNase A activity was measured by RT-qPCR targeting SARS-CoV-2 at different points in time as indicated (0–30 min post-spiking). Data are shown as a relative decrease in SARS-CoV-2 RNA (delta Ct) as compared to 0 min and are presented as a percentage.

To assess the influence of Tween-20 and Triton X-100 detergents, which are both commonly used for the inactivation of enveloped viruses, we spiked TBS buffer (0.9% NaCl, 50 mM Tris; pH: 8.5) with purified SARS-CoV-2 RNA. After spiking, we added either 0.1% Tween-20 or 0.5% Triton X-100 to the TBS buffer. As a next step, we added different concentrations of RNAse A (1 pg/mL, 0.1 pg/mL and 0.01 pg/mL) to the spiked TBS buffers. RT-qPCR revealed that Triton X-100 led to a rapid decline of RNA stability, independent of the RNAse A concentration applied (Figure 4). In comparison with TBS only, TBS with Tween-20 increased RNAse A activity, particularly at concentrations of 1 pg/mL and 0.1 pg/mL. In summary, both detergents increased RNA degradation.

### 3.5. SSB-4M Efficiently Inactivates SARS-CoV-2 Viral Particles

To be able to conduct a cell-based readout of virus infectivity in the presence of the cytotoxic reagents Triton X-100 and GuHCl, experiments were conducted with 10% SSB-4M diluted either with 0.9% NaCl or Tris/EDTA, which was subjected to size-exclusion chromatography after SARS-CoV-2 spiking. Irrespective of the diluent used, SARS-CoV-2 was below the detection limit of the tissue culture infectious dose assay within 2 min after the virus spike; thus, SSB-4M efficiently inactivated SARS-CoV-2 viral particles. Taking into account the cumulative volume of all kinetic sampling points, as well as the bulk titration of the last kinetic sample, overall reduction factors of >5.4 log10 and >4.8 log10 were obtained (Table 4).

## 4. Discussion

Assuring RNA sample stability is highly relevant for infectious disease diagnostics when considering delays between sampling and the start of RNA extraction. Furthermore, prolonged RNA stability is important for follow-up analysis, such as screening for newly emerging virus variants. However, data regarding RNA stability in sampling solutions and viral diagnostics only recently became an issue because of the SARS-CoV-2 pandemic and, consequently, the huge amounts of samples to be analysed in laboratories worldwide [14,15].

Regarding SARS-CoV-2 laboratory diagnostics, the three most common and reliable sample types to be analysed are naso- and oropharyngeal swabs and pharyngeal lavage [16]. It has been demonstrated that endogenous RNases are detrimental to RNA stability during collection, storage and testing of pharyngeal lavage and saliva samples [17,18,19,20,21,22]. The two prominent RNases, EDN (RNase 2) and ECP (RNase 3), are found in saliva [23,24], with increased expression during illness and infection [25,26,27,28,29,30,31]. In addition to endogenous RNAses, exogenously introduced enzymes might lead to RNA sample degradation. Therefore, suitable measures are required to inhibit RNAse activity. Another issue in laboratory diagnostics is virus inactivation, which should minimize the risk for laboratory workers being infected during routine work. Therefore, efficient virus inactivation measures that ensure RNA stability, thus assuring the quality of the applied diagnostic methodology, are urgently needed.

In this study, we presented a sample storage buffer that contains GuHCl and is uncomplicated to manufacture. The chaotropic agent GuHCl was chosen due to its environmental non-toxicity and compatibility with extraction systems [32]. In addition, the Triton X-100 detergent, well-known to inactivate lipid-enveloped viruses, such as SARS-CoV-2 [33,34,35], is another component of this buffer. We showed that our SSB-4M buffer is compatible with commercially available and in-house GuHCl-based RNA extraction methods and allows for the detection of SARS-CoV-2 RNA without affecting the performance quality of the applied test system. We also showed that SSB-4M preserves SARS-CoV-2 RNA stability, even in the presence of 10 ng/mL RNAse A and in storage at room temperature. This is of particular interest, as a recent study conducted by Kim et al. revealed that the rates of false-negative SARS-CoV-2 RT-qPCR results were increased if samples were stored at room temperature or above for only one day [36]. Nevertheless, we suggest sample storage at low temperatures, as this supports RNA stability.

Our data further revealed that different sample types require different storage buffer conditions to guarantee efficient preservation of SARS-CoV-2 RNA stability. Whereas SSB-4M efficiently stabilized RNA in naso- and oropharyngeal swabs, pharyngeal lavage requires higher concentrations of GuHCl to achieve the same protective effect. Pharyngeal lavage is proposed as a good sampling type, as it can be easily performed at home and without medical training [37,38,39]. Whereas some studies have found poorer efficiency of lavage sampling [40], other found equivalent viral loads for lavage and nasopharyngeal swabs [38]. Because we obtained all sample types from the same individuals and sampling was conducted by trained personnel, we assume that viral loads in our samples are comparable. Our findings indicate that the concentration of GuHCl and the presence of Triton X-100 in SSB-4M might be sufficient to destroy SARS-CoV-2 viral envelopes but does not efficiently inhibit RNase activity in pharyngeal lavage samples, thus allowing for the degradation of the released viral RNA by endogenous RNAses. Therefore, we investigated the sole effect of Triton X-100 and Tween-20 detergents, which are both frequently used to destroy viral envelopes. Our data show that both detergents, if applied as the sole reagent in a buffer, substantially decrease the stability of SARS-CoV-2 RNA, most likely due to RNAses that can easily access viral RNA after degradation of the lipid envelope of the virus.

Virus inactivation with different lysis and sample buffers has recently been an issue, and insufficient SARS-CoV-2 inactivation has been observed [7,9,10,41,42,43,44]. We showed that using a diluted form of our SSB-4M buffer efficiently inactivated SARS-CoV-2 in a tissue culture infectious dose assay. This indicates that once a sample is stored in SSB-4M, the risk for SARS-CoV-2 transmission, e.g., during sample delivery or further sample processing, is minimized. Our data clearly demonstrate that two components are essential for an efficient sample storage buffer: first, a reagent that inactivates the virus and, second, a reagent that inhibits RNAse activity.

The main limitation of our study is that our buffer has only been tested with GuHCl-based test systems. To date, we do not know whether the SSB presented in this study is also compatible with non-GuHCl-based RT-qPCR screening methods. Furthermore, sampling might affect our findings, as there is an ongoing debate concerning the viral load in different sample types. Another limitation regarding virus inactivation testing is that virus inactivation likely proceeded for a few minutes after SEC due to residual amounts of Triton X-100 and GuHCl. This has to be taken into account for the interpretation of virus inactivation after 2 min.

In conclusion, our study presents an uncomplicated sample storage buffer that is compatible with commercial and in-house GuHCl-based RT-qPCR screening methods. It allows for the efficient inactivation of infectious SARS-CoV-2 viral particles and the simultaneous preservation of RNA stability. Based on the findings of this study, we suggest the use of the presented GuHCl sample storage buffer to support and improve SARS-CoV-2 laboratory diagnostics.

## Figures and Tables

**Table 1:** Storage sample buffers and their chemical composition.

Storage Sample Buffer (SSB)	Buffer Composition
SSB-4M	4 M Guanidine-Hydrochloride (GuHCl) 50 mM Tris-HCl (pH 6.5) 20 mM EDTA-Na (pH 6.5) 1% (*v*/*v*) Triton X-100
SSB-6M	6 M GuHCl 75 mM Tris-HCl (pH 6.5) 30 mM EDTA-Na (pH 6.5) 1.5 % (*v*/*v*) Triton X-100

**Table 2:** Compatibility of SSB-4M with different commercial kits and an in-house RNA extraction protocol. Data shown are mean Ct values of five independent samples with standard deviation.

Nucleic Acid Extraction Method	Kit-Provided Lysis Buffer + NaCl 1:1	Kit-Provided Lysis Buffer + SSB-4M 1:1	SSB-4M Only
Qiagen Viral RNA Mini Kit	28.99 ± 0.25	29.34 ± 0.31	30.67 ± 0.16
Promega LEV Buccal Swab Kit	26.61 ± 0.35	26.15 ± 0.19	26.13 ± 0.06
Zymo Viral DNA/RNA MagBead Kit	26.00 ± 0.13	25.83 ± 0.09	25.96 ± 0.12
In-house RNA extraction protocol	26.43 ± 0.03	26.26 ± 0.17	25.99 ± 0.25

**Table 3:** Comparison of two RNA extraction and RT-qPCR methods regarding the detection of SARS-CoV-2-positive samples stored in SSB-4M. Naso- and oropharyngeal swabs and pharyngeal lavage of 16 previously SARS-CoV-2-positive individuals were compared by applying our in-house RNA extraction and RT-qPCR, as well as nucleic acid extraction and RT-qPCR with the Cobas 8800 test system from Roche Diagnostics. Presented data show the number of correctly identified SARS-CoV-2-positive samples out of 16 samples investigated in total.

	In-House Extraction and RT-qPCR			Roche Cobas 8800 Extraction and RT-qPCR		
	SARS-CoV-2 E-Gen	SARS-CoV-2 RdRP-Gen	Total Detected	SARS-CoV-2 Target 1	SARS-CoV-2 Target 2	Total Detected
Nasopharyngeal swab	14/16	15/16	15/16	15/16	15/16	15/16
Oropharyngeal swab	14/16	16/16	16/16	15/16	16/16	16/16
Pharyngeal lavage	6/16	13/16	14/16	9/16	11/16	11/16

**Table 4:** Inactivation kinetics of SSB-4M concerning SARS-CoV-2. SSB-4M was diluted to 1:10 with 0.9% NaCl (Run 1) or TRIS/EDTA buffer (Run 2) and spiked at a ratio of 1:11 with SARS-CoV-2. Samples were drawn at the indicated times and immediately subjected to size-exclusion chromatography (PD, i.e., PD-10 protein-desalting columns) and titration to determine residual infectious virus (log_10_[TCID_50_/mL]; < : no residual infectivity detected). To lower the limit of detection, (i) the sample of the last kinetic sampling point (60 min) was diluted (1:10E1.5) and subjected to ‘bulk titration’ (sample St60 B1.5), and (ii) the cumulative volume of successive negative samples (CVNS) was taken into account. Spike control (SC PD) and hold control (HC PD) samples were derived from a separate vessel containing the respective diluent.

		Run 1–10% SSB-4M, Diluted with 0.9% NaCl log10		Run 2–10% SSB-4M, Diluted with TRIS/EDTA	
Sample	Incubation Time (min)	SARS-CoV-2 Titre log10 (TCID50/mL)	∆ to SC log10 (TCID50/mL)	SARS-CoV-2 Titre log10 (TCID50/mL)	∆ to SC log10 (TCID50/mL)
Pos. Control	n.a.	7.0	-	6.5	-
SC PD	n.a.	5.9	-	5.3	-
St2 PD	2	<1.6	>4.3	<1.6	>3.7
St5 PD	5	<1.6	>4.3	<1.6	>3.7
St10 PD	10	<1.6	>4.3	<1.6	>3.7
St30 PD	30	<1.6	>4.3	<1.6	>3.7
St60 PD	60	<1.6	>4.3	<1.6	>3.7
St60 B1.5	60	<0.7	>5.2	<0.7	>4.6
CVNS	n.a.	<0.5	>5.4	<0.5	>4.8
HC PD	60	5.6	-	5.1	-

## Data Availability

Not applicable.
